# Fixed point results for weak contractions in partially ordered *b*-metric space

**DOI:** 10.1186/s13104-021-05649-x

**Published:** 2021-07-08

**Authors:** N. Seshagiri Rao, K. Kalyani, K. Prasad

**Affiliations:** 1 Department of Applied Mathematics, School of Applied Natural Sciences, Adama Science and Technology University, Post Box No.1888, Adama, Ethiopia; 2 Department of Mathematics, Vignan’s Foundation for Science, Technology & Research, Vadlamudi, 522213 Andhra Pradesh India; 3 Department of Applied Mathematics, Koneru Lakshmaiah Educational Foundation, Vaddeswaram, Andhra Pradesh India

**Keywords:** $({\check{\psi }}, \hat{\eta })$-weak contraction, Fixed point, Coincidence and coupled coincidence points, Ordered *b*-metric space, 54H25, 47H10

## Abstract

**Objectives:**

We explore the existence of a fixed point as well as the uniqueness of a mapping in an ordered *b*-metric space using a generalized $({\check{\psi }}, \hat{\eta })$-weak contraction. In addition, some results are posed on a coincidence point and a coupled coincidence point of two mappings under the same contraction condition. These findings generalize and build on a few recent studies in the literature. At the end, we provided some examples to back up our findings.

**Result:**

In partially ordered *b*-metric spaces, it is discussed how to obtain a fixed point and its uniqueness of a mapping, and also investigated the existence of a coincidence point and a coupled coincidence point for two mappings that satisfying generalized weak contraction conditions.

## Introduction

In a wide range of pure and applied mathematics problems, fixed points of mappings that satisfy contractive conditions in extended metric spaces are extremely useful. First, Ran and Reuings [32] described the existence of fixed points in this direction for certain maps in ordered metric space and exhibited matrix linear equations applications. Following that, Nieto et al. [28, 29] expanded the result of [32] to nondecreasing mappings and used their findings to obtain differential equations solutions. Agarwal et al. [3] and O’Regan et al. [30] examined the influence of generalized contractions in ordered spaces at the same time. Bhaskar and Lakshmikantham [11] first developed coupled fixed point theory for some maps, then used the results to find a unique solution to periodic boundary value problems. Following that, Lakshmikantham and Ćirić [22], which were the extensions of [11] involving monotone property to a function in the space, pioneered the idea of coupled coincidence, common fixed point results. [19, 25, 34–37] provide additional information on coupled fixed point effects in various spaces under various contractive conditions.

A *b*-metric space is one of several generalizations of a standard metric space proposed by Bakhtin in his work [9], and widely used by Czerwik in his work [14, 15]. Following that, a lot of progress was made in acquiring the results of fixed points to single valued as well as multi-valued operators in the space, as evidenced by [1, 2, 4–8, 10, 13, 16–18, 20, 21, 23, 24, 26, 27, 31, 38–41].

We demonstrate some fixed points results for mappings in ordered *b*-metric space that satisfy a generalized weak contraction in this paper. The results from [10, 11, 19, 22, 33] are expanded here as well as some examples noted to support the findings at the end of our work.

## Preliminaries

The following definitions are subsequently used in our study.

### Definition 2.1

[15] A *b*-metric is a mapping $\eth : \mathscr {E} \times \mathscr {E} \rightarrow [0, +\infty )$ that satisfies the properties below for all $\varepsilon ,\wp ,\zeta$ in $\mathscr {E}$ and some $\mathrm {s} \ge 1$,

- $\eth (\varepsilon ,\wp )=0$ if and if $\varepsilon =\wp$,
- $$\eth (\varepsilon ,\wp )=\eth (\wp ,\varepsilon )$$,
- $$\eth (\varepsilon ,\wp ) \le \mathrm {s} \left( \eth (\varepsilon ,\zeta )+\eth (\zeta ,\wp )\right) $$.

A *b*-metric space is specified as $(\mathscr {E},\eth ,\mathrm {s})$.

### Example 2.2

The space $L_q[0,1]$, where $0<q<1$ of all real functions $f(t), t \in [0,1]$ such that $\int _{0}^{1}|f(t)|^qdt<\infty$ is a *b*-metric space if we take $\eth (\varepsilon ,\wp )=\int _{0}^{1}(|f(t)-g(t)|^qdt)^{\frac{1}{q}}$, for all $\varepsilon ,\wp \in L_q[0,1]$.

### Note 2.3

Every metric space is a *b*-metric space with $\mathrm {s}=1$, but in general a *b*-metric space need not necessarily be a metric space, as in below example 2.4 is *b*-metric space but not a metric space. Thus, the class of *b*-metric spaces is larger than the class of metric spaces.

### Example 2.4

Let $\mathscr {E}=\mathbb {R}$ and define the mapping $\eth :\mathscr {E} \times \mathscr {E} \rightarrow \mathbb {R}^+$ by $\eth (\varepsilon ,\wp )=\left| \varepsilon -\wp \right| ^2$, for all $\varepsilon ,\wp \in \mathscr {E}$. Then $(\mathscr {E},\eth )$ is a *b*-metric space with coefficient $\mathrm {s}=2$.

The generalization of the above Example 2.4 is as follows:

### Example 2.5

Let $({\mathscr {E}},d)$ be a metric space and $q\ge 1$ be a given real number. Then $\eth (\varepsilon ,\wp )=\left[ d(\varepsilon ,\wp )\right] ^q$ is a *b*-metric on $\mathscr {E}$ with parameter $\mathrm {s}\le 2^{q-1}$.

### Definition 2.6

[10, 15] In a *b*-metric space,

1. if $\eth (\varepsilon _n,\varepsilon )\rightarrow 0$ as $n \rightarrow +\infty$ then $\{\varepsilon _n \}$ is said to be convergent to $\varepsilon$.
2. if $\eth (\varepsilon _n,\varepsilon _m) \rightarrow 0$ as $n,m \rightarrow +\infty$ then $\{\varepsilon _n\}$ is a Cauchy sequence.
3. if $(\mathscr {E},\eth ,\mathrm {s})$ is a complete *b*-metric space then very Cauchy sequence is convergent.

### Definition 2.7

[15, 33] If $\mathscr {E}$ is a partial ordered set with respect to an ordered relation $\preceq$ and $\eth$ is a metric on it, then $({\mathscr {E}},\eth ,\preceq )$ is a partially ordered metric space. $({\mathscr {E}},\eth ,\preceq )$ is complete partially ordered *b*-metric space, despite the fact that $\eth$ is complete.

### Definition 2.8

[33] Let ${\mathscr {h}}: {\mathscr {E}} \rightarrow {\mathscr {E}}$ be a mapping. If ${\mathscr {h}}(\varepsilon )\preceq {\mathscr {h}}(\wp )$ for all $\varepsilon ,\wp \in {\mathscr {E}}$ with $\varepsilon \preceq \wp$, then ${\mathscr {h}}$ is called monotone nondecreasing mapping.

### Definition 2.9

[12] Let ${\mathscr {h}},{\mathscr {I}}: {\mathscr {A}}\rightarrow {\mathscr {A}}$ be two mappings, and ${\mathscr{A}}\ne \emptyset \subseteq \mathscr {E}$. If ${\mathscr {h}}\varepsilon ={\mathscr {I}}\varepsilon =\varepsilon ~({\mathscr {h}}\varepsilon ={\mathscr {I}}\varepsilon )$ for $\varepsilon \in {\mathscr {A}}$, then $\varepsilon$ is called a common fixed point (coincidence point) of ${\mathscr {h}}$ and ${\mathscr {I}}$.

### Definition 2.10

[12] If ${\mathscr {h}}{\mathscr {I}}\varepsilon ={\mathscr {I}}{\mathscr {h}}\varepsilon$ for all $\varepsilon \in {\mathscr {A}}$, then ${\mathscr {h}}$ and ${\mathscr {I}}$ are commuting.

### Definition 2.11

[12, 33] The two self mappings ${\mathscr {h}}$ and ${\mathscr {I}}$ are known to be compatible, if $\lim \limits _{n \rightarrow +\infty } d({\mathscr {I}}{\mathscr {h}}\varepsilon _n,{\mathscr {h}}{\mathscr {I}}\varepsilon _n) =0$ for every sequence $\{\varepsilon _n\}$ in ${\mathscr {E}}$ such that $\lim \limits _{n \rightarrow +\infty }{\mathscr {h}}\varepsilon _n= \lim \limits _{n \rightarrow +\infty }{\mathscr {I}}{\mathscr {\varepsilon }}_n =\mu ,~ \text {for some}~ \mu \in {\mathscr {A}}$.

### Definition 2.12

[12, 33] If ${\mathscr {h}}\varepsilon ={\mathscr {I}}\varepsilon$ for some $\varepsilon \in {\mathscr {A}}$, then ${\mathscr {h}}{\mathscr {I}}\varepsilon ={\mathscr {I}}{\mathscr {h}}\varepsilon$, the mappings ${\mathscr {h}}$ and ${\mathscr {I}}$ are called weakly compatible.

### Definition 2.13

[33] If ${\mathscr {h}}\varepsilon \preceq {\mathscr {h}}\wp$ implies ${\mathscr {I}}\varepsilon \preceq {\mathscr {I}}\wp$ for each $\varepsilon ,\wp \in {\mathscr {E}}$, then the mapping ${\mathscr {I}}$ is called monotone ${\mathscr {h}}$-nondecreasing.

### Definition 2.14

[11] Let ${\mathscr {I}}: {\mathscr {E}} \times {\mathscr {E}} \rightarrow {\mathscr {E}}$ and ${\mathscr {h}}: {\mathscr {E}} \rightarrow {\mathscr {E}}$ are two mappings,

- a point $(\varepsilon ,\wp ) \in {\mathscr {E}} \times {\mathscr {E}}$ is coupled coincidence point of ${\mathscr {I}}$ and ${\mathscr {h}}$, if ${\mathscr {I}}(\varepsilon ,\wp )={\mathscr {h}}\varepsilon$ and ${\mathscr {I}}(\wp ,\varepsilon )={\mathscr {h}}\wp$. In particular, if ${\mathscr {h}}$ is an identity mapping, then $(\varepsilon ,\wp )$ is a coupled fixed point of ${\mathscr {I}}$.
- a point $\varepsilon \in {\mathscr {E}}$ is a common fixed point of ${\mathscr {I}}$ and ${\mathscr {h}}$, if ${\mathscr {I}}(\varepsilon ,\varepsilon )={\mathscr {h}}\varepsilon =\varepsilon$.
- if ${\mathscr {I}}({\mathscr {h}}\varepsilon ,{\mathscr {h}}\wp )={\mathscr {h}}({\mathscr {I}}\varepsilon ,{\mathscr {I}}\wp )$ for all $\varepsilon , \wp \in {\mathscr {E}}$, then ${\mathscr {I}}$ and ${\mathscr {h}}$ are commuting each other.
- If every two elements of ${\mathscr {A}} \subseteq {\mathscr {E}}$ are comparable, then the set ${\mathscr {A}}$ is called a well ordered set.

### Definition 2.15

A self mapping $\check{\psi }$ on $[0, +\infty )$ that meets the conditions below is known as an altering distance function:

- $\check{\psi }$ is a non-decreasing and continuous function,
- $\check{\psi }(\ell )=0$ if and only if $\ell =0$.

As seen above, the symbol $\hat{\Phi }$ represents the set of all altering distance functions.

Similarly, $$\hat{\Psi }: \{\hat{\eta }|\hat{\eta }~ {is ~a ~lower ~semi{\text{-}}continuous~ self~ mapping~ on}~[0, +\infty )~ \text {and,}~ \hat{\eta }(\ell )=0~ \text {if and only if}~ \ell =0\}$$.

The presented lemmas under here are frequently used in our main results.

### Lemma 2.16

[27] Let ${\mathscr {h}}: {\mathscr {E}}\rightarrow {\mathscr {E}}$ be a mapping, and ${\mathscr {E}}\ne \emptyset$. Then ${\mathscr {M}} \subseteq {\mathscr {E}}$ occurs, resulting in ${\mathscr {h}}{\mathscr {M}}={\mathscr {h}}{\mathscr {E}}$, where ${\mathscr {h}}:{\mathscr {M}} \rightarrow {\mathscr {E}}$ is one-to-one.

### Lemma 2.17

[4] Let $\{\varepsilon _n \}$ and $\{\wp _n\}$ be two sequences and *b*-convergent to $\varepsilon$ and $\wp$ in a *b*-metric space $({\mathscr {E}},\eth ,\mathrm {s},\preceq )$, where $\mathrm {s}>1$. Then

$$\begin{aligned} \frac{1}{\mathrm {s}^2}\eth (\varepsilon ,\wp )\le \lim \limits _{n \rightarrow +\infty } \inf \eth (\varepsilon _n,\wp _n) \le \lim \limits _{n \rightarrow +\infty } \sup \eth (\varepsilon _n,\wp _n)\le \mathrm {s}^2 \eth (\varepsilon ,\wp ). \end{aligned}$$

In particular, if $\varepsilon =\wp$, then $\lim \limits _{n \rightarrow +\infty } \eth (\varepsilon _n,\wp _n)=0$. In addition, for every $\tau \in \mathscr {E}$, we get

$$\begin{aligned} \frac{1}{\mathrm {s}}\eth (\varepsilon ,\tau )\le \lim \limits _{n \rightarrow +\infty } \inf \eth (\varepsilon _n,\tau ) \le \lim \limits _{n \rightarrow +\infty } \sup \eth (\varepsilon _n,\tau )\le \mathrm {s} d(\varepsilon ,\tau ). \end{aligned}$$

## Main results

We start this section with the following fixed point theorem in an ordered *b*-metric space.

### Theorem 3.1

Suppose $(\mathscr {E},\eth ,\mathrm {s},\preceq )$ is a complete partially ordered *b*-metric space with $\mathrm {s} > 1$. A mapping ${\mathscr {I}}:{\mathscr {E}} \rightarrow {\mathscr {E}}$ is continuous and nondecreasing with respect to $\preceq$. If $\varepsilon _0 \in {\mathscr {E}}$ is such that $\varepsilon _0 \preceq {\mathscr {I}}\varepsilon _0$ and the following contraction condition is fulfilled, then ${\mathscr {I}}$ has a fixed point in ${\mathscr {E}}$.

1 $$\begin{aligned} \check{\psi }({\mathrm {s}}\eth ({\mathscr {I} }\varepsilon ,{\mathscr {I}} \wp ))\le \check{\psi }({\mathscr {P}}(\varepsilon ,\wp ))-{\hat{\eta }}({\mathscr {P}}(\varepsilon ,\wp )), \end{aligned}$$

for $\check{\psi } \in \hat{\Phi }, \hat{\eta } \in \hat{\Psi }$ and for any $\varepsilon ,\wp \in {\mathscr {E}}$ such that $\varepsilon \preceq \wp$ and where

2 $$\begin{aligned} \mathscr {P}(\varepsilon ,\wp )=\max \left\{ \frac{\eth (\wp ,\mathscr {I}\wp ) \left[ 1+\eth (\varepsilon ,\mathscr {I}\varepsilon )\right] }{1+\eth (\varepsilon ,\wp )},\frac{\eth (\varepsilon ,\mathscr {I}\wp )+\eth (\wp ,\mathscr {I}\varepsilon )}{2s}, \eth (\varepsilon ,\mathscr {I}\varepsilon ),\eth (\wp ,\mathscr {I}\wp ),\eth (\varepsilon ,\wp )\right\} . \end{aligned}$$

### Proof

For some $\varepsilon _0 \in \mathscr {E}$ with $\mathscr {I}\varepsilon _0=\varepsilon _0$, then the result is trivial. Assuming that $\varepsilon _0 \prec \mathscr {I}\varepsilon _0$, we describe a sequence $\{\varepsilon _n\} \subset \mathscr {E}$ by $\varepsilon _{n+1}=\mathscr {I}\varepsilon _n$ for all $n\ge 0$. However, we can deduce the following as $\mathscr {I}$ is nondecreasing,

3 $$\begin{aligned} \varepsilon _0 \prec \mathscr {I}\varepsilon _0=\varepsilon _1\preceq \mathscr {I}\varepsilon _1=\varepsilon _2\preceq ...\preceq \mathscr {I}\varepsilon _{n-1}=\varepsilon _n \preceq \mathscr {I}\varepsilon _n=\varepsilon _{n+1}\preceq .....~. \end{aligned}$$

If $\varepsilon _{n_0}=\varepsilon _{n_0+1}$ for $n_0\in \mathbb {N}$, then $\varepsilon _{n_0}$ is a fixed point of $\mathscr {I}$ from (3). Otherwise, for all $n \ge 1$, $\varepsilon _n \ne \varepsilon _{n+1}$. For $n \ge 1$, let $D_n=\eth (\varepsilon _{n+1},\varepsilon _n)$. We know that for every $n \ge 1$, $\varepsilon _{n-1}\prec \varepsilon _n$ and, then the equation (1) becomes

4 $$\begin{aligned} \begin{aligned} \check{\psi }(D_n)=\check{\psi }(\eth (\varepsilon _n,\varepsilon _{n+1}))&= \check{\psi }(\eth (\mathscr {I}\varepsilon _{n-1},\mathscr {I}\varepsilon _n))\le \check{\psi }(\mathrm {s}\eth (\mathscr {I}\varepsilon _{n-1},\mathscr {I}\varepsilon _n))\\ {}&\le \check{\psi }(\mathscr {P}(\varepsilon _{n-1},\varepsilon _n))-\hat{\eta }(\mathscr {P}(\varepsilon _{n-1},\varepsilon _n)). \end{aligned} \end{aligned}$$

From (4), we get

5 $$\begin{aligned} \eth (\varepsilon _n,\varepsilon _{n+1})= \eth (\mathscr {I}\varepsilon _{n-1},\mathscr {I}\varepsilon _n)\le \frac{1}{\mathscr {s}} \mathscr {P}(\varepsilon _{n-1},\varepsilon _n), \end{aligned}$$

where

6 $${\mathscr{P}}({\varepsilon _{n - 1}},{\varepsilon _n}) = \max \left\{ {\frac{{\eth({\varepsilon _n},{\mathscr{I}}{\varepsilon _n})\left[ {1 + \eth({\varepsilon _{n - 1}},{\mathscr{I}}{\varepsilon _{n - 1}})} \right]}}{{1 + \eth({\varepsilon _{n - 1}},{\varepsilon _n})}},\frac{{\eth({\varepsilon _{n - 1}},{\mathscr{I}}{\varepsilon _n}) + \eth({\varepsilon _n},{\mathscr{I}}{\varepsilon _{n - 1}})}}{{2s}},{\mkern 1mu} \eth({\varepsilon _{n - 1}},{\mathscr{I}}{\varepsilon _{n - 1}}),\eth({\varepsilon _n},{\mathscr{I}}{\varepsilon _n}),\eth({\varepsilon _{n - 1}},{\varepsilon _n})} \right\} \leqslant \max \left\{ {\eth({\varepsilon _n},{\varepsilon _{n + 1}}),\frac{{\eth({\varepsilon _{n - 1}},{\varepsilon _n}) + \eth({\varepsilon _n},{\varepsilon _{n + 1}})}}{2},\eth({\varepsilon _{n - 1}},{\varepsilon _n})} \right\} \leqslant \max \{ {D_n},{D_{n - 1}}\} .$$

If $\max \{D_n,D_{n-1}\}= D_n$ for certain $n \ge 1$, equation (5) is then accompanied by

$$\begin{aligned} \eth (\varepsilon _n,\varepsilon _{n+1})\le \frac{1}{\mathscr {s}} \eth (\varepsilon _n,\varepsilon _{n+1}), \end{aligned}$$

this is a contradiction. Thus, $\max \{D_n,D_{n-1}\}= D_{n-1}$ for $n \ge 1$. Hence, equation (5) becomes

$$\begin{aligned} \eth (\varepsilon _n,\varepsilon _{n+1})\le \frac{1}{\mathscr {s}} \eth (\varepsilon _n,\varepsilon _{n-1}). \end{aligned}$$

Since $\frac{1}{\mathscr {s}}\in (0,1)$, then $\{\varepsilon _n\}$ is a Cauchy sequence from [1, 6, 8, 18]. Also, the completeness of $\mathscr {E}$ gives that $\varepsilon _n \rightarrow \mu \in \mathscr {E}$.

We may also deduce the following from the continuity of $\mathscr {I}$,

7 $$\begin{aligned} \mathscr {I}\mu =\mathscr {I}(\lim \limits _{n\rightarrow +\infty }\varepsilon _n)=\lim \limits _{n\rightarrow +\infty }\mathscr {I}\varepsilon _n=\lim \limits _{n\rightarrow +\infty }\varepsilon _{n+1}=\mu . \end{aligned}$$

As a result, $\mathscr {I}$ in $\mathscr {E}$ has a fixed point $\mu$. $\square$

The continuity assumption on $\mathscr {I}$ is extracted from Theorem 3.1 and used to derive the following theorem.

### Theorem 3.2

In Theorem 3.1, if $\mathscr {E}$ satisfies below condition, then $\mathscr {I}$ has a fixed point.

8 $$\begin{aligned} &{\text {If a non-decreasing sequence}} \  \{\varepsilon _n\} \subseteq { {\mathscr{E}}} \ {\text{and}}\, \varepsilon _n\rightarrow \sigma \ {\text {then}} \ \varepsilon _n \le \sigma ,\\ &{\text {for each}} \ n \in {\mathbb {N}},  \ {\text {i.e.,}} \ \sigma =\sup \varepsilon _n. \end{aligned} $$

### Proof

We have an increasing sequence $\{\varepsilon _n\} \subseteq \mathscr {E}$ that eventually converges to some $\sigma \in \mathscr {E}$ as a result of Theorem 3.1. But by the hypotheses for all *n*, $\varepsilon _n \preceq \sigma$, which means that $\sigma =\sup \varepsilon _n$.

We can now assert that $\sigma$ is a fixed point of $\mathscr {I}$. Assume that $\mathscr {I}\sigma \ne \sigma$. Let

9 $$\mathscr{P}(\varepsilon _{n} ,\sigma ) = \max \left\{ {\frac{{\eth(\sigma ,\mathscr{I}\sigma )\left[ {1 + \eth(\varepsilon _{n} ,{\mathscr{I}}\varepsilon _{n} )} \right]}}{{1 + \eth(\varepsilon _{n} ,\sigma )}},\frac{{\eth(\varepsilon _{n} ,{\mathscr{I}}\sigma ) + \eth(\sigma ,{\mathscr{I}}\varepsilon _{n} )}}{{2s}},\,\eth(\varepsilon _{n} ,{\mathscr{I}}\varepsilon _{n} ),\eth(\sigma ,{\mathscr{I}}\sigma ),\eth(\varepsilon _{n} ,\sigma )} \right\} $$

then taking limit as $n\rightarrow +\infty$ in the equation (9) and making use of $\lim \limits _{n\rightarrow +\infty }\varepsilon _n=\sigma$, we get

10 $$\begin{aligned} \lim \limits _{n \rightarrow +\infty }\mathscr {P}(\varepsilon _n, \sigma )= \max \{\eth (\sigma ,\mathscr {I}\sigma ),0\}=\eth (\sigma ,\mathscr {I}\sigma ). \end{aligned}$$

Since, $\varepsilon _n \preceq \sigma$ for each *n*, then we obtain the following from equations (1) and (9)

11 $$\begin{aligned} \check{\psi }(\eth (\varepsilon _{n+1}, \mathscr {I}\sigma ))=\check{\psi }(\eth (\mathscr {I}\varepsilon _n, \mathscr {I}\sigma ))\le \check{\psi }(s \eth (\mathscr {I}\varepsilon _n, \mathscr {I}\sigma ))\le \check{\psi }(\mathscr {P}(\varepsilon _n, \sigma ))-\hat{\eta }(\mathscr {P}(\varepsilon _n, \sigma )). \end{aligned}$$

Take limit as $n \rightarrow +\infty$ in (11) and from equation (10) as well as the properties of $\check{\psi }$, $\hat{\eta }$, we have

12 $$\begin{aligned} \check{\psi }(\eth (\sigma ,\mathscr {I}\sigma )) \le \check{\psi }(\eth (\sigma ,\mathscr {I}\sigma ))-\hat{\eta }(\eth (\sigma ,\mathscr {I}\sigma ))< \check{\psi }(\eth (\sigma ,\mathscr {I}\sigma )). \end{aligned}$$

This is a contradiction to $\mathscr {I}\sigma \ne \sigma$. Hence, $\mathscr {I}\sigma =\sigma$. $\square$

In the above theorems, the fixed point is unique if $\mathscr {E}$ meets the following condition.

13 $$\begin{aligned} \text {There exists a}~\sigma ~ \text {in}~ \mathscr {E}~ \text {that is comparable to}~ \varepsilon ~ \text {and}~ \wp ,~ \text {for each} ~\varepsilon ,\wp \in \mathscr {E}. \end{aligned}$$

### Theorem 3.3

If $\mathscr {E}$ assumes the condition (13) in Theorem 3.1 & 3.2, then $\mathscr {I}$ has a unique fixed point in $\mathscr {E}$.

### Proof

Theorems 3.1 & 3.2 show that the set of fixed points of $\mathscr {I}$ is nonempty. Assume $\varepsilon ^*\ne \wp ^*$ are fixed points of $\mathscr {I}$ to ensure uniqueness. Following that,

14 $$\begin{aligned} \check{\psi }(\eth (\mathscr {I}\varepsilon ^*, \mathscr {I}\wp ^*)) \le \check{\psi }(s\eth (\mathscr {I}\varepsilon ^*, \mathscr {I}\wp ^*)) \le \check{\psi }(\mathscr {P}(\varepsilon ^*, \wp ^*))-\hat{\eta }(\mathscr {P}(\varepsilon ^*, \wp ^*)), \end{aligned}$$

where

15 $${\mathscr{P}}(\varepsilon ^{*} ,\wp ^{*} ) = \max \left\{ {\frac{{\eth(\wp ^{*} ,{\mathscr{I}}\wp ^{*} )\left[ {1 + \eth(\varepsilon ^{*} ,{\mathscr{I}}\varepsilon ^{*} )} \right]}}{{1 + \eth(\varepsilon ^{*} ,\wp ^{*} )}},\,\frac{{\eth(\varepsilon ^{*} ,{\mathscr{I}}\wp ^{*} ) + \eth(\wp ^{*} ,{\mathscr{I}}\varepsilon ^{*} )}}{{2s}},\,\eth(\varepsilon ^{*} ,{\mathscr{I}}\varepsilon ^{*} ),\eth(\wp ^{*} ,{\mathscr{I}}\wp ^{*} ),\eth(\varepsilon ^{*} ,\wp ^{*} )} \right\}.{\text{ }} $$

Therefore from equations (14) and (15), we have

16 $$\begin{aligned} \check{\psi }(\eth (\varepsilon ^*, \wp ^*))=\check{\psi }(\eth (\mathscr {I}\varepsilon ^*, \mathscr {I}\wp ^*)) \le \check{\psi }(\eth (\varepsilon ^*, \wp ^*))-\hat{\eta }(\eth (\varepsilon ^*, \wp ^*))< \check{\psi }(\eth (\varepsilon ^*, \wp ^*)), \end{aligned}$$

this contradicts to $\varepsilon ^*\ne \wp ^*$. Hence, $\varepsilon ^*= \wp ^*$. $\square$

Now, we have the below corollary from Theorems 3.1 to 3.3.

### Corollary 3.4

Let $(\mathscr {E},\eth ,\preceq )$ be a partially ordered *b*-metric space. Suppose the mappings $\mathscr {I},\mathscr {h}: \mathscr {E} \rightarrow \mathscr {E}$ are continuous such that

$$(C_1)$$.
  17 $$\begin{aligned} \check{\psi }({\mathrm {s}} \eth ({\mathscr {I}}\varepsilon ,{\mathscr {I}}\wp ))\le \check{\psi }(\mathscr {P}_{\mathscr {h}}(\varepsilon ,\wp ))-\hat{\eta }({\mathscr {P}}_{\mathscr {h}}(\varepsilon ,\wp )), \end{aligned}$$
  for every $\varepsilon , \wp$ $\in {\mathscr {E}}$ with $\mathscr {h}\varepsilon \preceq \mathscr {h}\wp$, $\mathrm {s}>1$, $\check{\psi } \in \hat{\Phi }$, $\hat{\eta } \in \hat{\Psi }$ and, where
  18 $${\mathscr{P}}_{h} (\varepsilon ,\wp ) = \max \left\{ {\frac{{\eth(h\wp ,{\mathscr{I}}\wp )\left[ {1 + \eth(h\varepsilon ,{\mathscr{I}}\varepsilon )} \right]}}{{1 + \eth(h\varepsilon ,h\wp )}},\frac{{\eth(h\varepsilon ,{\mathscr{I}}\wp ) + \eth(h\wp ,{\mathscr{I}}\varepsilon )}}{{2s}},\eth(h\varepsilon ,{\mathscr{I}}\varepsilon ),\eth(h\wp ,{\mathscr{I}}\wp ),\eth(h\varepsilon ,h\wp )} \right\}.{\text{ }} $$
$$(C_2)$$.
$\mathscr {I}\mathscr {E} \subset \mathscr {h}\mathscr {E}$ and $\mathscr {h}\mathscr {E} \subseteq \mathscr {E}$ is complete,
$$(C_3)$$.
$\mathscr {I}$ is monotone $\mathscr {h}$-non-decreasing and
$$(C_4)$$.
$\mathscr {I}$ and $\mathscr {h}$ are compatible.

If for some $\varepsilon _0 \in \mathscr {E}$ such that $\mathscr {h}\varepsilon _0 \preceq \mathscr {I}\varepsilon _0$, then a pair of mappings $(\mathscr {I},\mathscr {h})$ has a coincidence point in $\mathscr {E}$.

### Proof

By Lemma 2.16, there exists $\mathscr {M} \subset \mathscr {E}$ such that $\mathscr {h} \mathscr {M} = \mathscr {h}\mathscr {E}$ and $\mathscr {h}: \mathscr {M} \rightarrow \mathscr {E}$ is one-to-one. Now define a map $\mathscr {k}: \mathscr {h}\mathscr {M} \rightarrow \mathscr {h}\mathscr {M}$ by $\mathscr {k}(\mathscr {h}\varepsilon )=\mathscr {I}\varepsilon$, $\varepsilon \in \mathscr {M}$. Since $\mathscr {h}$ is one-to-one on $\mathscr {M}$, $\mathscr {k}$ is well defined. Then, $\mathscr {h} \mathscr {M} = \mathscr {h}\mathscr {E}$ is complete and then (17) becomes

19 $$\begin{aligned} \check{\psi }(s \eth (\mathscr {k}(\mathscr {h}\varepsilon ),\mathscr {k}(\mathscr {h}\wp )))\le \check{\psi }(\mathscr {P}_\mathscr {h}(\varepsilon ,\wp ))-\hat{\eta }(\mathscr {P}_\mathscr {h}(\varepsilon ,\wp )), \end{aligned}$$

for every $\varepsilon$,$\wp$ $\in \mathscr {E}$ with $\mathscr {h}\varepsilon \preceq \mathscr {h}\wp$ and, where

20 $$ {\mathscr{P}}_{h} (\varepsilon ,\wp ) = \max \left\{ {\frac{{\eth(h\wp ,k\eth(h\wp ))\left[ {1 + \eth(h\varepsilon ,k\eth(h\varepsilon ))} \right]}}{{1 + \eth(h\varepsilon ,h\wp )}},\frac{{\eth(h\varepsilon ,k\eth(h\wp )) + \eth(h\wp ,k\eth(h\varepsilon ))}}{{2s}},\,\eth(h\varepsilon ,k\eth(h\varepsilon )),\eth(h\wp ,k\eth(h\wp )),\eth(h\varepsilon ,h\wp )} \right\}. $$

Let $\varepsilon _0 \in \mathscr {M}$ such that $\mathscr {h}\varepsilon _0 \preceq \mathscr {I}\varepsilon _0=\mathscr {k}(\mathscr {h}\varepsilon _0)$. Choose $\varepsilon _1 \in \mathscr {M}$ such that $\mathscr {h}\varepsilon _1 = \mathscr {I}\varepsilon _0=\mathscr {k}(\mathscr {h}\varepsilon _0)$. By continuing this process, we obtain a sequence $\{\mathscr {h}\varepsilon _n\} \subset \mathscr {h}\mathscr {M}$ such that $\mathscr {h}\varepsilon _{n+1}=\mathscr {I}\varepsilon _n=\mathscr {k}(\mathscr {h}\varepsilon _n)$ for $n\ge 0$. By using the similar argument as in the proof of Theorem 3.1, we obtain that $\{\mathscr {h}\varepsilon _n\} \subset \mathscr {h}\mathscr {M}$ is a *b*-Cauchy sequence. Since $\mathscr {h}\mathscr {M}$ is complete, there exists $\mathscr {v} \in \mathscr {h}\mathscr {M}$ such that $\lim \limits _{n \rightarrow +\infty }\mathscr {h}\varepsilon _n=\mathscr {v} \in \mathscr {h}\mathscr {E}$. Then

$$\begin{aligned} \lim \limits _{n \rightarrow +\infty }\mathscr {h}\varepsilon _n=\lim \limits _{n \rightarrow +\infty }\mathscr {I}\varepsilon _{n-1}=\mathscr {v}. \end{aligned}$$

From the condition $(C_4)$, we have

21 $$\begin{aligned} \lim \limits _{n \rightarrow +\infty }\eth (\mathscr {h}(\mathscr {I}\varepsilon _n), \mathscr {I}(\mathscr {h}\varepsilon _n))=0. \end{aligned}$$

Furthermore, the triangular inequality of *b*-metric, we have

22 $$\begin{aligned} \eth (\mathscr {I}\mathscr {v},\mathscr {h}\mathscr {v})\le s\eth (\mathscr {I}\mathscr {v},\mathscr {I}(\mathscr {h}\varepsilon _n))+s^2 \eth (\mathscr {I}(\mathscr {h}\varepsilon _n), \mathscr {h}(\mathscr {I}\varepsilon _n))+s^2\eth (\mathscr {h}(\mathscr {I}\varepsilon _n), \mathscr {h}\mathscr {v}). \end{aligned}$$

Taking $n \rightarrow +\infty$ in (22) and the continuity of $\mathscr {I}$, $\mathscr {h}$ and (21), we get $\eth (\mathscr {I}\mathscr {v},\mathscr {h}\mathscr {v})=0$. That is $\mathscr {I}\mathscr {v}=\mathscr {h}\mathscr {v}$. Therefore, $\mathscr {v}$ is a coincidence point of $\mathscr {I}$, $\mathscr {h}$.

The following result can get from Corollary 3.4 by weakening its hypotheses.

### Corollary 3.5

If $\mathscr {E}$ satisfies the following condition in Corollary 3.4,

23 $$\begin{aligned} &{\text{for very nondecreasing sequence}} \ \{\mathscr{h}\varepsilon _n\} \subseteq {\mathscr{E}} \ {\text{such that}} \ {\mathscr{h}}\varepsilon _n\rightarrow {\mathscr{h}}\sigma , \ {\text{then}} \\ {}&{\mathscr{h}}\varepsilon _n \le {\mathscr{h}}\sigma ~ (n \ge 0),\ {\text{i.e.,}} \ {\mathscr{h}}\sigma =\sup {\mathscr{h}}\varepsilon _n. \end{aligned}$$

then, if $\mathscr {h}\mu \preceq \mathscr {h}(\mathscr {h}\mu )$ for some coincidence point $\mu$, a coincidence point exists for the weakly compatible mappings $(\mathscr {I}, \mathscr {h})$. Moreover, $(\mathscr {I}, \mathscr {h})$ has only one common fixed point if and only if the set of common fixed points is well ordered. $\square$

### Proof

A pair of mappings $(\mathscr {I}, \mathscr {h})$ has a coincidence point, according to Theorem 3.3 and Corollary 3.4.

Next, assume that a pair of mappings $(\mathscr {I}, \mathscr {h})$ is weakly compatible. Let $\mathscr {v}\in \mathscr {E}$ be a point with $\mathscr {v}=\mathscr {I}\mu =\mathscr {h}\mu$. Then, $\mathscr {I}\mathscr {v}=\mathscr {I}(\mathscr {h}\mu )=\mathscr {h}(\mathscr {I}\mu )=\mathscr {h} \mathscr {v}$.

Therefore,

24 $$   {\mathscr{P}}_{\mathscr{h}} (\mu ,v) = \max \left\{ {\frac{{\eth(hv,Iv)\left[ {1 + \eth(h\mu ,I\mu )} \right]}}{{1 + \eth(h\mu ,hv)}},\frac{{\eth(h\mu ,Iv) + \eth(hv,I\mu )}}{{2s}},\,\,\eth(h\mu ,I\mu ),\eth(hv,Iv),\eth(h\mu ,hv)} \right\} = \max \left\{ {0,\frac{{\eth(I\mu ,Iv)}}{s},\eth(I\mu ,Iv)} \right\} = \eth(I\mu ,Iv).{\text{ }}  $$

Thus from equation (17), we get

25 $$\begin{aligned} \begin{aligned} \check{\psi }(\eth (\mathscr {I}\mu ,\mathscr {I}\mathscr {v})) \le \check{\psi }(\mathscr {P}_\mathscr {h}(\mu ,\mathscr {v}))-\hat{\eta }(\mathscr {P}_\mathscr {h} (\mu , \mathscr {v})) \le \check{\psi }(\eth (\mathscr {I}\mu ,\mathscr {I}\mathscr {v}))-\hat{\eta }(\eth (\mathscr {I}\mu ,\mathscr {I}\mathscr {v})). \end{aligned} \end{aligned}$$

By the property of $\hat{\eta }$, we get $\eth (\mathscr {I}\mu ,\mathscr {I}\mathscr {v})=0$ implies that $\mathscr {I}\mathscr {v}=\mathscr {h}\mathscr {v}=\mathscr {v}$.

Finally, we can deduce from Theorem 3.3 that $(\mathscr {I}, \mathscr {h})$ has only one common fixed point if and only if the common fixed points of $(\mathscr {I}, \mathscr {h})$ is well ordered. ${\square}$

### Remark 3.6

Theorems 3.1 to 3.3 are respectively the extension of Theorems 2.1,.2.2 & 2.3 of [27].

### Remark 3.7

Corollaries 3.4 & 3.5 are the generalizations of Corollaries 2.1 & 2.2 of [12] respectively.

### Definition 3.8

Consider a partially ordered *b*-metric space, $(\mathscr {E},\eth ,\preceq )$. A mapping $\mathscr {I}:\mathscr {E} \times \mathscr {E} \rightarrow \mathscr {E}$ is known to be a generalized $(\check{\psi },\hat{\eta })$-contractive mapping with regards to $\mathscr {h}:\mathscr {E} \rightarrow \mathscr {E}$, if

26 $$\begin{aligned} \check{\psi }(s^k\eth (\mathscr {I}(\varepsilon ,\wp ),\mathscr {I}(\zeta ,\mathfrak {I})))\le \check{\psi }(\mathscr {P}_\mathscr {h}(\varepsilon ,\wp ,\zeta ,\mathfrak {I}))-\hat{\eta }(\mathscr {P}_\mathscr {h}(\varepsilon ,\wp ,\zeta ,\mathfrak {I})), \end{aligned}$$

for all $\varepsilon ,\wp ,\zeta ,\mathfrak {I}\in \mathscr {E}$ with $\mathscr {h}\varepsilon \preceq \mathscr {h} \zeta$ and $\mathscr {h}\wp \succeq \mathscr {h} \mathfrak {I}$, $k>2$, $s>1$, $\check{\psi } \in \hat{\Phi }$, $\hat{\eta } \in \hat{\Psi }$ and where

$$ {\mathscr{P}}_{h} (\varepsilon ,\wp ,\zeta ,\Im ) = \max \left\{ {\frac{{\eth(h\zeta ,{\mathscr{I}}\eth(\zeta ,\Im ))\left[ {1 + \eth(h\varepsilon ,{\mathscr{I}}\eth(\varepsilon ,\wp ))} \right]}}{{1 + \eth(h\varepsilon ,h\zeta )}},\frac{{\eth(h\varepsilon ,{\mathscr{I}}\eth(\zeta ,\Im )) + \eth(h\zeta ,{\mathscr{I}}\eth(\varepsilon ,\wp ))}}{{2s}},\,\eth(h\varepsilon ,{\mathscr{I}}\eth(\varepsilon ,\wp )),\eth(h\zeta ,{\mathscr{I}}\eth(\zeta ,\Im )),\eth(h\varepsilon ,h\zeta )} \right\} $$

### Theorem 3.9

Suppose that $(\mathscr {E},\eth ,\preceq )$ is a complete partially ordered *b*-metric space. A mapping $\mathscr {I}:\mathscr {E} \times \mathscr {E} \rightarrow \mathscr {E}$ satisfies the condition (26) and $\mathscr {I}$, $\mathscr {h}$ are continuous, $\mathscr {I}$ has mixed $\mathscr {h}$-monotone property and also commutes with $\mathscr {h}$. Assume that, if for some $(\varepsilon _0,\wp _0) \in \mathscr {E} \times \mathscr {E}$ such that $\mathscr {h}\varepsilon _0 \preceq \mathscr {I}(\varepsilon _0,\wp _0)$, $\mathscr {h}\wp _0 \succeq \mathscr {I}(\wp _0,\varepsilon _0)$ and $\mathscr {I}(\mathscr {E} \times \mathscr {E}) \subseteq \mathscr {h}(\mathscr {E})$, then $\mathscr {I}$ and $\mathscr {h}$ have a coupled coincidence point in $\mathscr {E}$.

### Proof

From Theorem 2.2 of [7], there exist two sequences $\{\varepsilon _n\}$ and $\{\wp _n\}$ in $\mathscr {E}$ such that

$$\begin{aligned} \mathscr {h}\varepsilon _{n+1}=\mathscr {I}(\varepsilon _n,\wp _n), ~~\mathscr {h}\wp _{n+1}=\mathscr {I}(\wp _n,\varepsilon _n), n\ge 0. \end{aligned}$$

In particular, the sequences $\{\mathscr {h}\varepsilon _n\}$ and $\{\mathscr {h}\wp _n\}$ are non-decreasing and non-increasing in $\mathscr {E}$. Put $\varepsilon =\varepsilon _n, \wp =\wp _n, \zeta =\varepsilon _{n+1}, \mathfrak {I}=\wp _{n+1}$ in (26), we get

27 $$\begin{aligned} \begin{aligned} \check{\psi }(s^k\eth (\mathscr {h}\varepsilon _{n+1},\mathscr {h}\varepsilon _{n+2}))&=\check{\psi }(s^k\eth (\mathscr {I}(\varepsilon _n,\wp _n),\mathscr {I}(\varepsilon _{n+1},\wp _{n+1})))\\ {}&\le \check{\psi }(\mathscr {P}_\mathscr {h}(\varepsilon _n,\wp _n,\varepsilon _{n+1},\wp _{n+1}))-\hat{\eta }(\mathscr {P}_\mathscr {h}(\varepsilon _n,\wp _n,\varepsilon _{n+1},\wp _{n+1})), \end{aligned} \end{aligned}$$

where

28 $$\begin{aligned} \mathscr {P}_\mathscr {h}(\varepsilon _n,\wp _n,\varepsilon _{n+1},\wp _{n+1})\le \max \{\eth (\mathscr {h}\varepsilon _n,\mathscr {h}\varepsilon _{n+1}),\eth (\mathscr {h}\varepsilon _{n+1},\mathscr {h}\varepsilon _{n+2})\}. \end{aligned}$$

Therefore from (27), we have

29 $$\begin{aligned} \begin{aligned} \check{\psi }(s^k\eth (\mathscr {h}\varepsilon _{n+1},\mathscr {h}\varepsilon _{n+2}))&\le \check{\psi }(\max \{\eth (\mathscr {h}\varepsilon _n,\mathscr {h}\varepsilon _{n+1}),\eth (\mathscr {h}\varepsilon _{n+1},\mathscr {h}\varepsilon _{n+2})\})\\ {}&~~~~~-\hat{\eta }(\max \{\eth (\mathscr {h}\varepsilon _n,\mathscr {h}\varepsilon _{n+1}),\eth (\mathscr {h}\varepsilon _{n+1},\mathscr {h}\varepsilon _{n+2})\}). \end{aligned} \end{aligned}$$

Similarly by taking $\varepsilon =\wp _{n+1}, \wp =\varepsilon _{n+1}, \zeta =\varepsilon _n, \mathfrak {I}=\varepsilon _n$ in (26), we get

30 $$\begin{aligned} \begin{aligned} \check{\psi }(s^k\eth (\mathscr {h}\wp _{n+1},\mathscr {h}\wp _{n+2}))&\le \check{\psi }(\max \{\eth (\mathscr {h}\wp _n,\mathscr {h}\wp _{n+1}),\eth (\mathscr {h}\wp _{n+1},\mathscr {h}\wp _{n+2})\})\\ {}&~~~~~~~-\hat{\eta }(\max \{\eth (\mathscr {h}\wp _n,\mathscr {h}\wp _{n+1}),\eth (\mathscr {h}\wp _{n+1},\mathscr {h}\wp _{n+2})\}). \end{aligned} \end{aligned}$$

We know that $\max \{\check{\psi }(l_1),\check{\psi }(l_2)\}=\check{\psi } \{\max \{l_1,l_2\}\}$ for $l_1,l_2 \in [0,+\infty )$. Then by adding (29) and (30) together we get,

31 $$\begin{aligned} \begin{aligned} \check{\psi }(s^k \Gamma _n)&\le \check{\psi }(\max \{\eth (\mathscr {h}\varepsilon _n,\mathscr {h}\varepsilon _{n+1}),\eth (\mathscr {h}\varepsilon _{n+1},\mathscr {h}\varepsilon _{n+2}),\eth (\mathscr {h}\wp _n,\mathscr {h}\wp _{n+1}),\eth (\mathscr {h}\wp _{n+1},\mathscr {h}\wp _{n+2})\})\\ {}&-\hat{\eta }(\max \{\eth (\mathscr {h}\varepsilon _n,\mathscr {h}\varepsilon _{n+1}),\eth (\mathscr {h}\varepsilon _{n+1},\mathscr {h}\varepsilon _{n+2}),\eth (\mathscr {h}\wp _n,\mathscr {h}\wp _{n+1}),\eth (\mathscr {h}\wp _{n+1},\mathscr {h}\wp _{n+2})\}), \end{aligned} \end{aligned}$$

where

32 $$\begin{aligned} \Gamma _n=\max \{\eth (\mathscr {h}\varepsilon _{n+1},\mathscr {h}\varepsilon _{n+2}), \eth (\mathscr {h}\wp _{n+1},\mathscr {h}\wp _{n+2})\}. \end{aligned}$$

Let us denote,

33 $$\begin{aligned} \varkappa _n=\max \{\eth (\mathscr {h}\varepsilon _n,\mathscr {h}\varepsilon _{n+1}),\eth (\mathscr {h}\varepsilon _{n+1},\mathscr {h}\varepsilon _{n+2}),\eth (\mathscr {h}\wp _n,\mathscr {h}\wp _{n+1}),\eth (\mathscr {h}\wp _{n+1},\mathscr {h}\wp _{n+2})\}. \end{aligned}$$

Hence from equations (29)-(32), we obtain

34 $$\begin{aligned} s^k\Gamma _n\le \varkappa _n. \end{aligned}$$

Now to claim that

35 $$\begin{aligned} \Gamma _n\le \lambda \Gamma _{n-1}, \end{aligned}$$

for $$n \ge 1$$ and $$\lambda =\frac{1}{s^k} \in [0,1)$$.

Suppose that if $\varkappa _n=\Gamma _n$ then from (34), we get $s^k\Gamma _n\le \Gamma _n$ this leads to $\Gamma _n=0$, since $s>1$ and thus (35) holds. Suppose $\varkappa _n=\max \{\eth (\mathscr {h}\varepsilon _n,\mathscr {h}\varepsilon _{n+1}), \eth (\mathscr {h}\wp _n,\mathscr {h}\wp _{n+1})\}$, i.e., $\varkappa _n=\Gamma _{n-1}$ then (34) follows (35).

Now from (34), we obtain that $\Gamma _n\le \lambda ^n \delta _0$ and hence,

36 $$\begin{aligned} \eth (\mathscr {h}\varepsilon _{n+1},\mathscr {h}\varepsilon _{n+2})\le \lambda ^n \Gamma _0 ~~\text {and}~~\eth (\mathscr {h}\wp _{n+1},\mathscr {h}\wp _{n+2})\le \lambda ^n \Gamma _0, \end{aligned}$$

which shows that $\{\mathscr {h}\varepsilon _n\}$ and $\{\mathscr {h}\wp _n\}$ in $\mathscr {E}$ are Cauchy sequences by Lemma 3.1 of [20]. Therefore, we can conclude from Theorem 2.2 of [5] that, $\mathscr {I}$ and $\mathscr {h}$ have a coincidence point in $\mathscr {E}$. $\square$

### Corollary 3.10

Suppose that $(\mathscr {E},\eth ,\preceq )$ is a complete partially ordered *b*-metric space. A continuous mapping $\mathscr {I}:\mathscr {E} \times \mathscr {E} \rightarrow \mathscr {E}$ has a mixed monotone property and is satisfying the below contraction conditions for all $\varepsilon ,\wp ,\zeta ,\mathfrak {I}\in \mathscr {E}$ such that $\varepsilon \preceq \zeta$ and $\wp \succeq \mathfrak {I}$, $k>2$, $s>1$, $\check{\psi } \in \hat{\Phi }$ and $\hat{\eta } \in \hat{\Psi }$:

(i). $$\begin{aligned} \check{\psi }(s^k\eth (\mathscr {I}(\varepsilon ,\wp ),\mathscr {I}(\zeta ,\mathfrak {I})))\le \check{\psi }(\mathscr {P}_\mathscr {h}(\varepsilon ,\wp ,\zeta ,\mathfrak {I}))-\hat{\eta }(\mathscr {P}_\mathscr {h}(\varepsilon ,\wp ,\zeta ,\mathfrak {I})), \end{aligned}$$
(ii). $$\begin{aligned} \eth (\mathscr {I}(\varepsilon ,\wp ),\mathscr {I}(\zeta ,\mathfrak {I}))\le \frac{1}{s^k}\mathscr {P}_\mathscr {h}(\varepsilon ,\wp ,\zeta ,\mathfrak {I})-\frac{1}{s^k}\hat{\eta }(\mathscr {P}_\mathscr {h}(\varepsilon ,\wp ,\zeta ,\mathfrak {I})), \end{aligned}$$

where

$$ {\mathscr{P}}_{} (\varepsilon ,\wp ,\zeta ,\Im ) = \max \left\{ {\frac{{\eth(\zeta ,{\mathscr{\mathscr{I}}}\eth(\zeta ,\Im ))\left[ {1 + \eth(\varepsilon ,{\mathscr{\mathscr{I}}}\eth(\varepsilon ,\wp ))} \right]}}{{1 + \eth(\varepsilon ,\zeta )}},\frac{{\eth(\varepsilon ,{\mathscr{\mathscr{I}}}\eth(\zeta ,\Im )) + \eth(\zeta ,{\mathscr{\mathscr{I}}}\eth(\varepsilon ,\wp ))}}{{2s}},~~\eth(\varepsilon ,{\mathscr{\mathscr{I}}}\eth(\varepsilon ,\wp )),\eth(\zeta ,{\mathscr{\mathscr{I}}}\eth(\zeta ,\Im )),\eth(\varepsilon ,\zeta )} \right\}.{\text{ }} $$

If there exists $(\varepsilon _0,\wp _0) \in \mathscr {E} \times \mathscr {E}$ such that $\varepsilon _0 \preceq \mathscr {I}(\varepsilon _0,\wp _0)$ and $\wp _0 \succeq \mathscr {I}(\wp _0,\varepsilon _0)$, then $\mathscr {I}$ has a coupled fixed point in $\mathscr {E}$.

### Theorem 3.11

The unique coupled common fixed point for $\mathscr {I}$ and $\mathscr {h}$ exists in Theorem 3.9, if for every $(\varepsilon ,\wp ),(\mathscr {k},\mathscr {l}) \in \mathscr {E} \times \mathscr {E}$ there exists some $(\Lambda ,\Upsilon )\in \mathscr {E} \times \mathscr {E}$ such that $(\mathscr {I}(\Lambda ,\Upsilon ), \mathscr {I}(\Upsilon ,\Lambda ))$ is comparable to $(\mathscr {I}(\varepsilon ,\wp ), \mathscr {I}(\wp ,\varepsilon ))$ and to $(\mathscr {I}(\mathscr {k},\mathscr {I}),\mathscr {I}(\mathscr {l},\mathscr {k}))$.

### Proof

The existence of a coupled coincidence point for $\mathscr {I}$ and $\mathscr {h}$ is guaranteed by the Theorem 3.9. Let $(\varepsilon , \wp ),(\mathscr {k},\mathscr {l}) \in \mathscr {E} \times \mathscr {E}$ are two coupled coincidence points of $\mathscr {I}$ and $\mathscr {h}$. Now, we assert that $\mathscr {h}\varepsilon =\mathscr {h}\mathscr {k}$ and $\mathscr {h}\wp =\mathscr {h}\mathscr {l}$. By the hypotheses $(\mathscr {I}(\Lambda ,\Upsilon ), \mathscr {I}(\Upsilon ,\Lambda ))$ is comparable to $(\mathscr {I}(\varepsilon ,\wp ), \mathscr {I}(\wp ,\varepsilon ))$ and to $(\mathscr {I}(\mathscr {k},\mathscr {I}),\mathscr {I}(\mathscr {l},\mathscr {k}))$ for some $(\Lambda ,\Upsilon )\in \mathscr {E} \times \mathscr {E}$.

Now, assume the following

$$\begin{aligned} (\mathscr {I}(\varepsilon ,\wp ), \mathscr {I}(\wp ,\varepsilon )) \le (\mathscr {I}(\Lambda ,\Upsilon ), \mathscr {I}(\Upsilon ,\Lambda )) ~\text {and}~ (\mathscr {I}(\mathscr {k},\mathscr {l}),\mathscr {I}(\mathscr {l},\mathscr {k}))\le (\mathscr {I}(\Lambda ,\Upsilon ), \mathscr {I}(\Upsilon ,\Lambda )). \end{aligned}$$

Suppose $\Lambda _0=\Lambda$ and $\Upsilon _0=\Upsilon$ then there is a point $(\Lambda _1,\Upsilon _1) \in \mathscr {E} \times \mathscr {E}$ such that

$$\begin{aligned} \mathscr {h}\Lambda _1=\mathscr {I}(\Lambda _0,\Upsilon _0),~~ \mathscr {h}\Upsilon _1=\mathscr {I}(\Upsilon _0,\Lambda _0)~~(n \ge 1). \end{aligned}$$

As by applying the preceding argument repeatedly, we have the sequences $\{\mathscr {h} \Lambda _{n}\}$ and $\{\mathscr {h} \Upsilon _{n}\}$ in $\mathscr {E}$ such that

$$\begin{aligned} \mathscr {h}\Lambda _{n+1}=\mathscr {I}(\Lambda _n,\Upsilon _n),~~ \mathscr {h}\Upsilon _{n+1}=\mathscr {I}(\Upsilon _n,\Lambda _n)~~(n \ge 0). \end{aligned}$$

Define the sequences in the same way $\{\mathscr {h} \varepsilon _{n}\}$, $\{\mathscr {h} \wp _{n}\}$ and, $\{\mathscr {h} \mathscr {k}_{n}\}$, $\{\mathscr {h} \mathscr {l}_{n}\}$ in $\mathscr {E}$ by setting $\varepsilon _0=\varepsilon$, $\wp _0=\wp$ and $\mathscr {k}_0=\mathscr {k}$, $\mathscr {l}_0=\mathscr {l}$. Further, we have that

37 $$\begin{aligned} \mathscr {h}\varepsilon _{n} \rightarrow \mathscr {I}(\varepsilon ,\wp ),~\mathscr {h}\wp _{n} \rightarrow \mathscr {I}(\wp ,\varepsilon ),~ \mathscr {h}\mathscr {k}_{n} \rightarrow \mathscr {I}(\mathscr {k},\mathscr {l}),~\mathscr {h}\mathscr {l}_n \rightarrow \mathscr {I}(\mathscr {l},\mathscr {k})~~(n \ge 1). \end{aligned}$$

Thus by induction, we get

38 $$\begin{aligned} (\mathscr {h}\varepsilon _{n},\mathscr {h}\wp _{n}) \le (\mathscr {h}\Lambda _n,\mathscr {h}\Upsilon _n)~~\text {for every}~n. \end{aligned}$$

As a consequence of (26), we have

39 $$\begin{aligned} \begin{aligned} \check{\psi }(\eth (\mathscr {h}\varepsilon ,\mathscr {h}\Lambda _{n+1}))\le \check{\psi }(s^k\eth (\mathscr {h}\varepsilon ,\mathscr {h}\Lambda _{n+1}))&= \check{\psi }(s^k\eth (\mathscr {I}(\varepsilon ,\wp ),\mathscr {I}(\Lambda _n,\Upsilon _n))) \\ {}&\le \check{\psi }(\mathscr {P}_\mathscr {h}(\varepsilon ,\wp ,\Lambda _n,\Upsilon _n))-\hat{\eta }(\mathscr {P}_\mathscr {h}(\varepsilon ,\wp ,\Lambda _n,\Upsilon _n)), \end{aligned} \end{aligned}$$

where

$${\mathscr{P}}_{h} (\varepsilon ,\wp ,\Lambda _{n} ,\Upsilon _{n} ) = \max \left\{ {\frac{{\eth(h\Lambda _{n} ,{\mathscr{I}}\eth(\Lambda _{n} ,\Upsilon _{n} ))\left[ {1 + \eth(h\varepsilon ,{\mathscr{I}}\eth(\varepsilon ,\wp ))} \right]}}{{1 + \eth(h\varepsilon ,h\Lambda _{n} )}},~\,\frac{{\eth(h\varepsilon ,{\mathscr{I}}\eth(\Lambda _{n} ,\Upsilon _{n} )) + \eth(h\Lambda _{n} ,{\mathscr{I}}\eth(\varepsilon ,\wp )}}{{2s}},\eth(h\varepsilon ,{\mathscr{I}}\eth(\varepsilon ,\wp )),\,\eth(h\Lambda _{n} ,{\mathscr{I}}\eth(\Lambda _{n} ,\Upsilon _{n} )),\eth(h\varepsilon ,h\Lambda _{n} )} \right\} = \max \left\{ {0,\frac{{\eth(h\varepsilon ,h\Lambda _{n} )}}{s},\,\eth(h\varepsilon ,h\Lambda _{n} )} \right\} = \eth(h\varepsilon ,h\Lambda _{n} ).{\text{ }} $$

Therefore from (39), we have

40 $$\begin{aligned} \check{\psi }(\eth (\mathscr {h}\varepsilon ,\mathscr {h}\Lambda _{n+1}))\le \check{\psi }(\eth (\mathscr {h}\varepsilon ,\mathscr {h}\Lambda _n))-\hat{\eta }(\eth (\mathscr {h}\varepsilon ,\mathscr {h}\Lambda _n)). \end{aligned}$$

As by the similar argument, we acquire that

41 $$\begin{aligned} \check{\psi }(\eth (\mathscr {h}\wp ,\mathscr {h}\Upsilon _{n+1}))\le \check{\psi }(\eth (\mathscr {h}\wp ,\mathscr {h}\Upsilon _n))-\hat{\eta }(\eth (\mathscr {h}\wp ,\mathscr {h}\Upsilon _n)). \end{aligned}$$

Hence from (40) and (41), we have

42 $$\begin{aligned} \begin{aligned} \check{\psi }(\max \{\eth (\mathscr {h}\varepsilon ,\mathscr {h}\Lambda _{n+1}),\eth (\mathscr {h}\wp ,\mathscr {h}\Upsilon _{n+1})\})&\le \check{\psi }(\max \{\eth (\mathscr {h}\varepsilon ,\mathscr {h}\Lambda _n),\eth (\mathscr {h}\wp ,\mathscr {h}\Upsilon _n)\})\\ {}&~~~~~~~-\hat{\eta }(\max \{\eth (\mathscr {h}\varepsilon ,\mathscr {h}\Lambda _n),\eth (\mathscr {h}\wp ,\mathscr {h}\Upsilon _n)\}) \\ {}&<\check{\psi }(\max \{\eth (\mathscr {h}\varepsilon ,\mathscr {h}\Lambda _n),\eth (\mathscr {h}\wp ,\mathscr {h}\Upsilon _n)\}). \end{aligned} \end{aligned}$$

Thus the property of $\check{\psi }$ implies,

$$\begin{aligned} \max \{\eth (\mathscr {h}\varepsilon ,\mathscr {h}\Lambda _{n+1}),\eth (\mathscr {h}\wp ,\mathscr {h}\Upsilon _{n+1})\} <\max \{\eth (\mathscr {h}\varepsilon ,\mathscr {h}\Lambda _n),\eth (\mathscr {h}\wp ,\mathscr {h}\Upsilon _n)\}. \end{aligned}$$

Hence, $\max \{\eth (\mathscr {h}\varepsilon ,\mathscr {h}\Lambda _n),\eth (\mathscr {h}\wp ,\mathscr {h}\Upsilon _n)\}$ is a decreasing sequence of positive reals and bounded below and by a result, we have

$$\begin{aligned} \lim \limits _{n \rightarrow +\infty }\max \{\eth (\mathscr {h}\varepsilon ,\mathscr {h}\Lambda _n),\eth (\mathscr {h}\wp ,\mathscr {h}\Upsilon _n)\} =\Gamma ,~\Gamma \ge 0. \end{aligned}$$

Therefore as $n \rightarrow +\infty$ in equation (42), we get

43 $$\begin{aligned} \check{\psi }(\Gamma )\le \check{\psi }(\Gamma )-\hat{\eta }(\Gamma ), \end{aligned}$$

from which we get $\hat{\eta }(\Gamma )=0$, this implies that $\Gamma =0$. Therefore,

$$\begin{aligned} \lim \limits _{n \rightarrow +\infty }\max \{\eth (\mathscr {h}\varepsilon ,\mathscr {h}\Lambda _n),\eth (\mathscr {h}\wp ,\mathscr {h}\Upsilon _n)\} =0. \end{aligned}$$

Hence, we have

44 $$\begin{aligned} \lim \limits _{n \rightarrow +\infty }\eth (\mathscr {h}\varepsilon ,\mathscr {h}\Lambda _n) =0 ~ \text {and} ~\lim \limits _{n \rightarrow +\infty }\eth (\mathscr {h}\wp ,\mathscr {h}\Upsilon _n) =0. \end{aligned}$$

From the similar argument as above, we obtain that

45 $$\begin{aligned} \lim \limits _{n \rightarrow +\infty }\eth (\mathscr {h}\mathscr {k},\mathscr {h}\Lambda _n) =0 ~ \text {and} ~\lim \limits _{n \rightarrow +\infty }\eth (\mathscr {h}\mathscr {I},\mathscr {h}\Upsilon _n) =0. \end{aligned}$$

Therefore from (44) and (45), we get $\mathscr {h}\varepsilon =\mathscr {h}\mathscr {k}$ and $\mathscr {h}\wp =\mathscr {h}\mathscr {I}$. Since $\mathscr {h}\varepsilon =\mathscr {I}(\varepsilon ,\wp )$ and $\mathscr {h}\wp =\mathscr {I}(\wp ,\varepsilon )$ and, the commutative property of $\mathscr {I}$ and $\mathscr {h}$ implies that

46 $$\begin{aligned} \mathscr {h}(\mathscr {h}\varepsilon )= \mathscr {h}(\mathscr {I}(\varepsilon ,\wp ))=\mathscr {I}(\mathscr {h}\varepsilon ,\mathscr {h}\wp )~ \text {and}~\mathscr {h}(\mathscr {h}\wp )= \mathscr {h}(\mathscr {I}(\wp ,\varepsilon ))=\mathscr {I}(\mathscr {h}\wp ,\mathscr {h}\varepsilon ). \end{aligned}$$

If $\mathscr {h}\varepsilon =\Lambda ^*$ and $\mathscr {h}\wp =\Upsilon ^*$, then from (46), we get

47 $$\begin{aligned} \mathscr {h}(\Lambda )= \mathscr {I}(\Lambda ^*,\Upsilon ^*)~ \text {and}~\mathscr {h}(\Upsilon ^*)= \mathscr {I}(\Upsilon ^*,\Lambda ^*), \end{aligned}$$

which exhibits that $(\Lambda ^*,\Upsilon ^*)$ is a coupled coincidence point of $\mathscr {I}$, $\mathscr {h}$. Hence, $\mathscr {h}(\Lambda ^*)=\mathscr {h}\mathscr {k}$ and $\mathscr {h}(\Upsilon ^*)=\mathscr {h}\mathscr {I}$ which in turn gives that $\mathscr {h}(\Lambda )=\Lambda ^*$ and $\mathscr {h}(\Upsilon ^*)=\Upsilon ^*$. Therefore from (47), $(\Lambda ^*,\Upsilon ^*)$ is a coupled common fixed point of $\mathscr {I}$, $\mathscr {h}$.

Let $(\Lambda _1^*,\Upsilon _1^*)$ be another coupled common fixed point of $\mathscr {I}$, $\mathscr {h}$. Then, $\Lambda _1^*=\mathscr {h}\Lambda _1^*= \mathscr {I}(\Lambda _1^*,\Upsilon _1^*)$ and $\Upsilon _1^*=\mathscr {h}\Upsilon _1^*= \mathscr {I}(\Upsilon _1^*,\Lambda _1^*)$. But $(\Lambda _1^*,\Upsilon _1^*)$ is a coupled common fixed point of $\mathscr {I}$ and $\mathscr {h}$ then, $\mathscr {h}\Lambda _1^*=\mathscr {h}\varepsilon =\Lambda$ and $\mathscr {h}\Upsilon _1^*=\mathscr {h}\wp =\Upsilon ^*$. Therefore, $\Lambda _1^*=\mathscr {h}\Lambda _1^*=\mathscr {h}\Lambda =\Lambda$ and $\Upsilon _1^*=\mathscr {h}\Upsilon _1^*=\mathscr {h}\Upsilon ^*=\Upsilon ^*$. Hence the uniqueness. $\square$

### Theorem 3.12

In Theorem 3.11, if $\mathscr {h}\varepsilon _0 \preceq \mathscr {h}\wp _0$ or $\mathscr {h}\varepsilon _0 \succeq \mathscr {h}\wp _0$, then a unique common fixed point of $\mathscr {I}$ and $\mathscr {h}$ can be found.

### Proof

Assume that $(\varepsilon ,\wp ) \in \mathscr {E}$ is a unique coupled common fixed point of $\mathscr {I}$ and $\mathscr {h}$. Then to demonstrate that $\varepsilon =\wp$. Suppose that $\mathscr {h}\varepsilon _0 \preceq \mathscr {h}\wp _0$, then we get by induction that, $\mathscr {h}\varepsilon _n \preceq \mathscr {h}\wp _n$ for $n \ge 0$. From Lemma 2 of [21], we have

$$\begin{aligned} \begin{aligned} \check{\psi }(s^{k-2}\eth (\varepsilon ,\wp ))&=\check{\psi }(s^k \frac{1}{s^2}\eth (\varepsilon ,\wp )) \le \lim \limits _{n \rightarrow +\infty }\sup \check{\psi }(s^k \eth (\varepsilon _{n+1}, \wp _{n+1})) \\ {}&= \lim \limits _{n \rightarrow +\infty }\sup \check{\psi }(s^k \eth (\mathscr {I}(\varepsilon _n, \wp _n),\mathscr {I}(\wp _n,\varepsilon _n))) \\ {}&\le \lim \limits _{n \rightarrow +\infty }\sup \check{\psi }(\mathscr {P}_\mathscr {h}(\varepsilon _n, \wp _n,\wp _n,\varepsilon _n))-\lim \limits _{n \rightarrow +\infty }\inf \hat{\eta }(\mathscr {P}_\mathscr {h}(\varepsilon _n, \wp _n,\wp _n,\varepsilon _n)) \\ {}&\le \check{\psi }(\eth (\varepsilon ,\wp ))-\lim \limits _{n \rightarrow +\infty }\inf \hat{\eta }(\mathscr {P}_\mathscr {h}(\varepsilon _n, \wp _n,\wp _n,\varepsilon _n)) \\ {}&<\check{\psi }(\eth (\varepsilon ,\wp )), \end{aligned} \end{aligned}$$

a contradiction. Hence, $$\varepsilon =\wp $$.

The result can also be similar in the case of $\mathscr {h}\varepsilon _0 \succeq \mathscr {h}\wp _0$. $\square$

### Remark 3.13

While $s=1$ and the result of [19], the condition

$$\begin{aligned} \check{\psi }(\eth (\mathscr {I}(\varepsilon ,\wp ),\mathscr {I}(\eth ,\mathfrak {I}))) \le \check{\psi }(\max \{\eth (\mathscr {h}\varepsilon ,\mathscr {h}\eth ),\eth (\mathscr {h}\wp ,\mathscr {h}\mathfrak {I})\})-\hat{\eta }(\max \{\eth (\mathscr {h}\varepsilon ,\mathscr {h}\eth ),\eth (\mathscr {h}\wp ,\mathscr {h}\mathfrak {I})\}) \end{aligned}$$

is equivalent to,

$$\begin{aligned} \eth (\mathscr {I}(\varepsilon ,\wp ),\mathscr {I}(\eth ,\mathfrak {I}))\le \varphi (\max \{\eth (\mathscr {h}\varepsilon ,\mathscr {h}\eth ),\eth (\mathscr {h}\wp ,\mathscr {h}\mathfrak {I})\}), \end{aligned}$$

where $\check{\psi } \in \hat{\Phi }$, $\hat{\eta } \in \hat{\Psi }$ and $\varphi$ is a continuous self mapping on $[0,+\infty )$ with $\varphi (y)<y$ for every $y>0$ with $\varphi (y)=0$ if and only if $y=0$. Hence the results found here are generalized and extended the results of [11, 18, 22, 25, 27] and several comparable results.

Now depending on the type of a metric, some examples are shown here under.

### Example 3.14

Let $\mathscr {E}=\mathscr {\{}e_1,e_2,e_3,e_4,e_5,e_6\}$ and $\eth :\mathscr {E} \times \mathscr {E} \rightarrow \mathscr {E}$ be a metric defined by

$$ (\varepsilon ,\wp ) = (\wp ,\varepsilon ) = 0,\ {\text{if}} \ \varepsilon  = \wp  = \{ e_{1} ,e_{2} ,e_{3} ,e_{4} ,e_{5} ,e_{6} \} \ {\text{and}} \ \varepsilon  = \wp , \ (\varepsilon ,\wp ) = (\wp ,\varepsilon ) = 3,\ if \ \varepsilon  = \wp  =  \{e_{1} ,e_{2} ,e_{3} ,e_{4} ,e_{5} \}  \ {\text{and}} \ \varepsilon  \ne \wp ,\ (\varepsilon ,\wp ) = (\wp ,\varepsilon ) = 12,\ if\ \varepsilon  = \{e_{1} ,e_{2} ,e_{3} ,e_{4} \} \ {\text{and}} \ \wp  = e_{6} ,\ (\varepsilon ,\wp ) = (\wp ,\varepsilon ) = 20,\ if\ \varepsilon  = e_{5} \ {\text{and}}\ \wp  = e_{6} ,\ {\text{with usual order}} \le .$$

A self-mapping $\mathscr {I}$ on $\mathscr {E}$ defined by $\mathscr {I}e_1=\mathscr {I}e_2=\mathscr {I}e_3=\mathscr {I}e_4=\mathscr {I}e_5=1, \mathscr {I}e_6=2$ has a fixed point with $\check{\psi }(y)=\frac{y}{2}$ and $\hat{\eta }(y)=\frac{y}{4}$ where $y \in [0,+\infty )$.

### Proof

When $s=2$, $(\mathscr {E},\eth ,\le )$ is a complete partially ordered *b*-metric space. Let $\varepsilon , \wp \in \mathscr {E}$ such that $\varepsilon < \wp$ then we’ll look at the cases below.

**Case 1**. If $\varepsilon , \wp \in \mathscr {\{}e_1,e_2,e_3,e_4,e_5\}$ then $\eth (\mathscr {I}\varepsilon ,\mathscr {I}\wp )=\eth ( e_1 , e_1 )=0$. Hence,

$$\begin{aligned} \check{\psi }(2\eth (\mathscr {I}\varepsilon ,\mathscr {I}\wp ))=0 \le \check{\psi }(\mathscr {P}(\varepsilon ,\wp ))-\hat{\eta }(\mathscr {P}(\varepsilon ,\wp )). \end{aligned}$$

**Case 2**. If $\varepsilon \in \mathscr {\{}e_1,e_2,e_3,e_4,e_5\}$ and $\wp = e_6$, then $\eth (\mathscr {I}\varepsilon ,\mathscr {I}\wp )=\eth ( e_1 , e_2 )=3$, $\mathscr {P}( e_6 , e_5 )=20$ and $\mathscr {P}(\varepsilon , e_6 )=12$, for $\varepsilon \in \mathscr {\{}e_1,e_2,e_3,e_4\}$. Hence,

$$\begin{aligned} \check{\psi }(2\eth (\mathscr {I}\varepsilon ,\mathscr {I}\wp )) \le \frac{\mathscr {P}(\varepsilon ,\wp )}{ 4 } =\check{\psi }(\mathscr {P}(\varepsilon ,\wp ))-\hat{\eta }(\mathscr {P}(\varepsilon ,\wp )). \end{aligned}$$

As a result, all of the conditions of Theorem 3.1 are met, and hence $\mathscr {I}$ has a fixed point. $\square$

### Example 3.15

Let us define a metric $\eth$ with usual order $\le$ by

$$\begin{aligned} \begin{aligned} \eth (\varepsilon ,\wp )= {\left\{ \begin{array}{ll} &{} ~0 ~~~~~~~~~,~~ if~ \varepsilon =\wp \\ &{} ~1 ~~~~~~~~~,~~ if~ \varepsilon \ne \wp \in \{0,1\} \\ &{} ~|\varepsilon -\wp |~~~,~~ if~ \varepsilon ,\wp \in \{0, \frac{1}{2n},\frac{1}{2m}: n \ne m \ge 1\} \\ &{} ~6 ~~~~~~~~~~,~~ otherwise. \end{array}\right. } \end{aligned} \end{aligned}$$

where $\mathscr {E}=\{0, 1, \frac{1}{2},\frac{1}{3},\frac{1}{4},...,\frac{1}{n},...\}$. A self-mapping $\mathscr {I}$ on $\mathscr {E}$ by $\mathscr {I}0=0, \mathscr {I}\frac{1}{n}=\frac{1}{12n} (n\ge 1)$ has a fixed point with $\check{\psi }(y)=y$ and $\hat{\eta }(y)=\frac{4y}{5}$ for $y \in [0,+\infty )$.

### Proof

$\eth$ is clearly discontinuous, and $(\mathscr {E},\eth ,\le )$ is a complete partially ordered *b*-metric space for $s=\frac{12}{5}$. Now we’ll look at the following cases for $\varepsilon ,\wp \in \mathscr {E}$ with $\varepsilon <\wp$.

**Case 1**. Suppose $\varepsilon =0$ and $\wp =\frac{1}{n} ~(n >0)$, then $\eth (\mathscr {I}\varepsilon ,\mathscr {I}\wp )=\eth (0,\frac{1}{12n})=\frac{1}{12n}$ and $\mathscr {P}(\varepsilon ,\wp )=\frac{1}{n}$ and $\mathscr {P}(\varepsilon ,\wp )= \{1,6\}$. Thus,

$$\begin{aligned} \check{\psi }\left( \frac{12}{5}\eth (\mathscr {I}\varepsilon ,\mathscr {I}\wp )\right) \le \frac{\mathscr {P}(\varepsilon ,\wp )}{5} =\check{\psi }(\mathscr {P}(\varepsilon ,\wp ))-\hat{\eta }(\mathscr {P}(\varepsilon ,\wp )). \end{aligned}$$

**Case 2**. Let $\varepsilon =\frac{1}{m}$ and $\wp =\frac{1}{n}$ where $m>n\ge 1$, then

$$\begin{aligned} \eth (\mathscr {I}\varepsilon ,\mathscr {I}\wp )=\eth (\frac{1}{12m},\frac{1}{12n}), \mathscr {P}(\varepsilon ,\wp )\ge \frac{1}{n}-\frac{1}{m}~ \text {or}~ \mathscr {P}(\varepsilon ,\wp )=6. \end{aligned}$$

Thus,

$$\begin{aligned} \check{\psi }\left( \frac{12}{5}\eth (\mathscr {I}\varepsilon ,\mathscr {I}\wp )\right) \le \frac{\mathscr {P}(\varepsilon ,\wp )}{5} =\check{\psi }(\mathscr {P}(\varepsilon ,\wp ))-\hat{\eta }(\mathscr {P}(\varepsilon ,\wp )). \end{aligned}$$

Hence, we have the conclusion from Theorem 3.1 as all assumptions are fulfilled. $\square$

### Example 3.16

Define a metric $d:\mathscr {E}\times \mathscr {E} \rightarrow \mathscr {E}$, where $\mathscr {E}=\{\tilde{\ell }/\tilde{\ell }:[a_1,a_2] \rightarrow [a_1,a_2]~ \text {is continuous}\}$ by

$$\begin{aligned} \eth (\tilde{\ell }_1,\tilde{\ell }_2)=\sup _{y \in [a_1,a_2]}\{| \tilde{\ell }_1(y)-\tilde{\ell }_2(y)|^2\} \end{aligned}$$

for any $\tilde{\ell }_1,\tilde{\ell }_2 \in \mathscr {E}$, $0 \le a_1<a_2$ with $\tilde{\ell }_1 \preceq \tilde{\ell }_2$ implies $a_1\le \tilde{\ell }_1(y) \le \tilde{\ell }_2 (y)\le a_2, y \in [a_1,a_2]$. A self-mapping $\mathscr {I}$ on $\mathscr {E}$ defined by $\mathscr {I} \tilde{\ell }= \frac{\tilde{\ell }}{5}, \tilde{\ell } \in \mathscr {E}$ has a unique fixed point with $\check{\psi }(y)=y$ and $\hat{\eta }(y)=\frac{y}{3}$ for any $y \in [0, +\infty ]$.

### Proof

As $\min (\tilde{\ell }_1,\tilde{\ell }_2) (y)=\min \{\tilde{\ell }_1(y),\tilde{\ell }_2(y)\}$ is continuous and all other assumptions of Theorem 3.3 are fulfilled for $s=2$. Hence, $0 \in \mathscr {E}$ is a unique fixed point of $\mathscr {I}$. $\square$

## Limitations

We examined a fixed point, a coincidence point and a couple coincidence point for mappings that are satisfying generalized $(\check{\psi }, \hat{\eta })$-weak contractions in a partially ordered *b*-metric space. The findings in this paper are generalized and extended a few well-known results in the current literature. Some examples are shown at the end to support the results obtained here.

## Data Availability

Not applicable.
